# The effect of phonics-enhanced Big Book reading on the language and literacy skills of 6-year-old pupils of different reading ability attending lower SES schools

**DOI:** 10.3389/fpsyg.2014.01222

**Published:** 2014-11-13

**Authors:** Laura Tse, Tom Nicholson

**Affiliations:** ^1^School of Curriculum and Pedagogy, The University of AucklandAuckland, New Zealand; ^2^Institute of Education, Massey UniversityAuckland, New Zealand

**Keywords:** spelling, phonemic awareness, reading comprehension, Big Book reading, phonics, achievement gap, shared book, math

## Abstract

The purpose of this study was to improve the literacy achievement of lower socioeconomic status (SES) children by combining explicit phonics with Big Book reading. Big Book reading is a component of the text-centered (or book reading) approach used in New Zealand schools. It involves the teacher in reading an enlarged book to children and demonstrating how to use semantic, syntactic, and grapho-phonic cues to learn to read. There has been little research, however, to find out whether the effectiveness of Big Book reading is enhanced by adding explicit phonics. In this study, a group of 96 second graders from three lower SES primary schools in New Zealand were taught in 24 small groups of four, tracked into three different reading ability levels. All pupils were randomly assigned to one of four treatment conditions: a control group who received math instruction, Big Book reading enhanced with phonics (BB/EP), Big Book reading on its own, and Phonics on its own. The results showed that the BB/EP group made significantly better progress than the Big Book and Phonics groups in word reading, reading comprehension, spelling, and phonemic awareness. In reading accuracy, the BB/EP and Big Book groups scored similarly. In basic decoding skills the BB/EP and Phonics groups scored similarly. The combined instruction, compared with Big Book reading and phonics, appeared to have no comparative disadvantages and considerable advantages. The present findings could be a model for New Zealand and other countries in their efforts to increase the literacy achievement of disadvantaged pupils.

## Introduction

The main reason for this study was to address the literacy needs of lower socioeconomic status (SES) pupils. These students start school with lower levels of pre-reading skills (Nicholson, 1997, 2003; Foster and Miller, 2007; Reardon, 2011, 2013), make slower gains in reading skills in their first years of school (Nicholson, 1997, 2003; Claessens et al., 2009), and make up more of those pupils who receive remedial tuition in Reading Recovery, 18% in lower SES schools as against 11% in higher SES schools (Cowles, 2013). Since this is the case, an important goal is to teach reading more effectively in lower SES schools so that pupils in those schools make more progress than they do at the moment.

The idea behind this study was to find out if the present text-centered or book reading approach used in most classrooms in New Zealand schools could be modified to increase its effectiveness. The text centered approach includes Big Book reading, reading of a wide range of graded readers, shared book reading with the teacher in small groups, as well as oral language and writing activities. One way to enhance the effectiveness of the text-centered approach would be to combine Big Book reading with explicit phonics to find out if this combination would be more effective in raising achievement than Big Book reading or phonics on its own. Enhancing Big Book reading with explicit phonics and phonemic awareness, both well known for their effectiveness (Gough, 1996; National Reading Panel, 2000; Ezell and Justice, 2005; Tunmer and Nicholson, 2011) could add an additional source of information to classroom instruction that helps disadvantaged pupils learn to read more quickly and increase their reading achievement.

### Big books

Big Book reading (Holdaway, 1982) is a technique to enable the teacher to interact with the class so that they pay more attention to text print as well as attending to illustrations and enjoying the story. Big Book reading involves enlarging the size of the reading material so that a whole class can see the print clearly and engage with it not just in terms of meaning but also in terms of looking at printed words and mentally figuring out how letters in words correspond to sounds in speech.

With Big Books (Ministry of Education, 2003) the teacher reads an enlarged copy of a graded reader so that a whole class can see the print clearly and engage with it not just in terms of meaning but also in terms of word reading. When Big Books first started, teachers made their own books, copying the text onto large pieces of paper but nowadays Big Books are produced commercially. Following the initial reading, pupils may re-read the Big Book with the teacher either that day or during later readings (Ministry of Education, 2003) based on the principle of teachers reading books to the class, then with the class, and finally the class reading the book by themselves.

The teacher reads the same book aloud to the class usually once a day from Monday to Thursday before moving to a new Big Book the following week. To encourage pupils to focus more on the text and less on illustrations, the teacher, while reading to beginner pupils, often follows the line of text with their finger or with a pointer and stops the reading at times to explain language features including unfamiliar vocabulary, punctuation (such as upper case letters or speech marks), or to discuss with the class some decoding aspect of the text, such as a consonant blend.

A feature of Big Book reading is that it does not teach explicit phonics. Pupils learn phonological recoding implicitly and incidentally in the context of reading. The teacher points out letter sound relationships, e.g., *sun* starts with s but phonological recoding is not taught explicitly as in “s-u-n.” Instead the teacher usually encourages pupils to use the initial letter or letters of the word plus sentence cues or illustrations to work out the unfamiliar word. Big Book reading therefore does not teach phonics as sounding out words in full as most phonics handbooks suggest (for example, see Nicholson, 2005) but it does encourage use of initial letter sounds and consonant blends (e.g., gr, st, sp) in conjunction with other contextual cues to predict unknown words without focusing on letter-by-letter sounding out. In this way, pupils are given hints as to how to decode words with phonics but are not directly taught to sound out the entire word (Ministry of Education, 2003). The theory is that pupils use the initial letters of the word plus contextual cues and illustrations to work out the meaning of the word but as they continue with reading of Big Books they will infer the phonological rules of decoding especially through acquisition of sub-lexical knowledge through frequent exposure to text. For a review of the acquisition of word reading and implicit phonological recoding in a text-centered way of teaching reading, see Fletcher-Flinn and Thompson (2010) and Thompson (2014).

### Vocabulary

Big Book reading also seems to improve vocabulary. Students learn new words when listening to stories (Elley and Mangubhai, 1983; Nicholson and Dymock, 2010). They also learn words when reading stories on their own (Suggate et al., 2013). There are individual differences in vocabulary learning from Big Book reading in that there are greater vocabulary gains for those pupils who are from higher socioeconomic (SES) backgrounds (McBride-Chang, 2012; Reese, 2012), or who have higher initial vocabulary knowledge (Robbins and Ehri, 1994), or who are better readers (Nicholson and Whyte, 1992).

### Phonics and phonemic awareness instruction

The value of phonemic awareness and phonics instruction is well known. The results of meta-analyses indicate that phonemic awareness (Bus and Ijzendoorn, 1999; Ehri et al., 2001b) and phonics are effective especially for lower SES pupils (National Reading Panel, 2000; Ehri et al., 2001a; Jeynes, 2008; Hattie, 2009; Suggate, 2010; Arrow and Tunmer, 2012).

A theoretical rationale for teaching phonemic awareness and phonics is code-cipher theory. Gough and Hillinger (1980) argued that beginner readers will learn to read if they have: (a) alphabet knowledge, (b) phonemic awareness, (c) cipher intent, that is, where the pupil attempts to recode letters in words according to their phonemes, and (d) data, that is, printed-spoken pairings of words where the pupil sees the word and hears it at the same time. Phonemic awareness and phonics instruction provides a, b, and c and may provide d if the teacher uses text material for pupils to read. Big Book reading definitely provides d but a, b, and c are not taught explicitly so that pupils who lack skill in these areas may not learn to read as quickly as those who have these skills when they start school, skills that higher SES pupils do tend to have more of when they begin school (Nicholson, 2003).

### Combined instruction

Pressley (2006) has been an influential voice in favor of balanced reading instruction that combines text centered reading instruction (including Big Books) with phonics and phonemic awareness skills. To illustrate the value of balanced instruction, Pressley made an analogy with two different ways of training children to play little league baseball. Learning to read with the book reading approach would be like training for Little League only by playing games. The downside of learning to play baseball by playing games is that if pupils go into games not knowing the skills of how to grip a bat, how to connect with the ball, or what direction to run, then playing games will not make them better players. On the other hand, training for Little League only by practicing batting, fielding, and running will not help unless pupils get a chance to play real baseball games. A little league player will do better with a combined training strategy, that is, by learning skills and then applying them in match practice.

### Reading ability

The present study took reading ability into account in that previous researchers have found that the effects of reading programs are different depending on reading ability. Juel and Minden-Cupp (2000) and Connor et al. (2004) found that the impact of the classroom reading program depended on the reading level of the pupil in that pupils with lower levels of decoding skill did better with a phonics emphasis while pupils who had higher levels of decoding skills did better in classrooms that had a text-centered reading focus.

### Aims

In the present study the benefit of balanced instruction was tested empirically by comparing Big Book reading on its own, phonics on its own, and Big Book reading enhanced with phonics (BB/EP). Pressley (2006) and Pressley and Fingeret (2007) argue that text-centered reading instruction and explicit phonics on their own are not enough and that balanced instruction is more likely to benefit pupils yet there is little research that directly compares a combination of Big Book reading and phonics with Big Book reading and phonics on their own. The present study aimed to fill this gap.

The aims were twofold, first to discover whether enhancing Big Book reading with phonics and phonemic awareness activities leads to measurable improvements in reading, spelling, phonemic awareness, and receptive vocabulary over and beyond that achieved with either Big Book reading or phonics on their own, and second, to measure whether phonics-enhanced Big Book reading achieves greater changes across different levels of reading ability compared with phonics and Big Book reading on their own.

Thus, there were two research questions:

1. Would a group of children who received combined instruction (BB/EP) make more progress than a group who received only Big Book reading, a group who received only explicit phonics, or a control group who received only math instruction?
2. Would the effects of each reading treatment, BB/EP, Big Book (BB) reading, and explicit phonics (P) vary for children with different levels of reading ability?

## Materials and methods

### Participants

Participants were 96 grade 2 (6-year-old) pupils who attended three low- SES primary schools in South Auckland, New Zealand (children in New Zealand start school when they turn five). The schools in the study had a decile 1 rating which is the lowest SES classification (Norris et al., 1994) used by the Ministry of Education in New Zealand. The Ministry uses census data to rank schools on a 1–10 basis (called deciles) based on SES related variables, such as household incomes and occupation of parents. Schools in the lowest categories receive government assistance.

There were 55 boys and 41 girls. Average age at the start of the study was 6 years and 3 months. Nearly all pupils in the study were Maori (42.7%) or from the Pacific Islands (56.3%). English-only was spoken at home by nearly half of the participants (46.9%). Other languages spoken at home in addition to English were Maori (15.6%), Pacific Island languages (36.5%), and for one child, Vietnamese (1%). None of the students received Reading Recovery tuition during the study, which is individual reading tuition available from the government for 6-year-old students not responding to the regular classroom program. All students had already completed a year of formal reading instruction.

### Research design and procedure

#### Design

The research plan employed a mixed factorial design. The between-subjects factors included two fixed-effect factors, Ability (High, Middle and Low), and Treatment (Combined, Phonics, Big Book, and Math Control). Within each of these combinations were two Teaching Groups of four students each. Teaching Group is a random-effect factor nested within Ability and Treatment, with Students a random-effect factor nested within Teaching Group. The between-subjects design is shown in Table 1. Pre-Post was a repeated-measure factor crossed with the between-subjects design.

**Table 1:** **Design of the experiment showing the number of children in each subgroup according to conditions and level of reading ability (*N* = 96)**.

**Reading Ability**	**Teaching subgroups**	**Conditions**				**Total *n* = 96**
		**Control (Math) *n* = 24**	**Big Book and phonics *n* = 24**	**Big Book *n* = 24**	**Phonics *n* = 24**	
Higher	Group1	4	4	4	4	32
	Group2	4	4	4	4	
Middle	Group3	4	4	4	4	32
	Group4	4	4	4	4	
Lower	Group5	4	4	4	4	32
	Group6	4	4	4	4	
Total		24	24	24	24	96

#### Procedure

The 96 students were divided into three ability groups based on their scores on the Burt Word Reading Test (Gilmore et al., 1981). Within each ability group, pupils were randomly assigned to four treatment groups: Combined (BB/EP), Big Book only, Phonics only, and Control (this group received alternative instruction in math). Within each treatment-by-ability combination, pupils were divided into two teaching groups.

There was no difference in chronological age among the four treatment groups. Chronological age for each group was: Combined (6.29 years), Phonics (6.28 years), Big Book (6.25 years), and Control (6.31 years), *F*_(3, 92)_ = 0.27, *p* > 0.05.

Burt word reading ages for the three ability groups were: higher (6.46 years); middle (5.75 years); lower (5.29 years). Only students in the higher ability group were reading at their chronological age.

All students completed pretest and posttest assessments of word reading, reading accuracy, reading comprehension, basic word decoding skills, phonemic awareness, receptive vocabulary, word spelling and math computation. One of the authors administered all the assessments. It took 4 weeks to complete the pre-assessments in May and 5 weeks to complete the post-assessments in November. All scoring was cross-checked with another marker until there was 100% agreement.

Teaching interventions ran for 12 weekly sessions, with one 30-min lesson each week taught to each of the 24 subgroups of four students, a total of 24 lessons per week. The tutor always taught the students in small groups of four. As each subgroup consisted of students with either lower, middle, or higher reading ability levels, the phonics lesson plan, the Big Books, and the Math exercises were different for each ability level. All groups received the same amount of time for instruction.

At the end of the study each subgroup had received 12 lessons. There were two school holidays during the training period (a total of 4 weeks) which lengthened the intervention time period.

**Figure 1:**
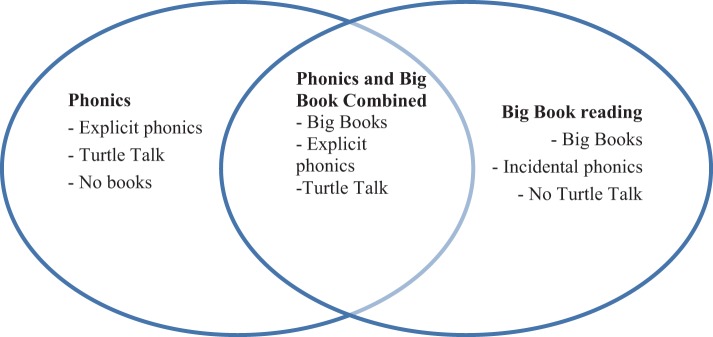
**Diagram of differences among phonics, Big Book, and combined (BB/EP) strategies**.

Within each of the four training groups, there were three different levels of ability for reading or math and each of these subgroups received a different package of lessons. The Phonics group worked on a different phonics rule each week. The Big Books group worked on four Big Books over the 12 lessons, re-reading each Big Book across three lessons. The first author was the tutor for all lessons. Figure 1 shows the differences in instruction for the three reading groups.

**Figure 2:**
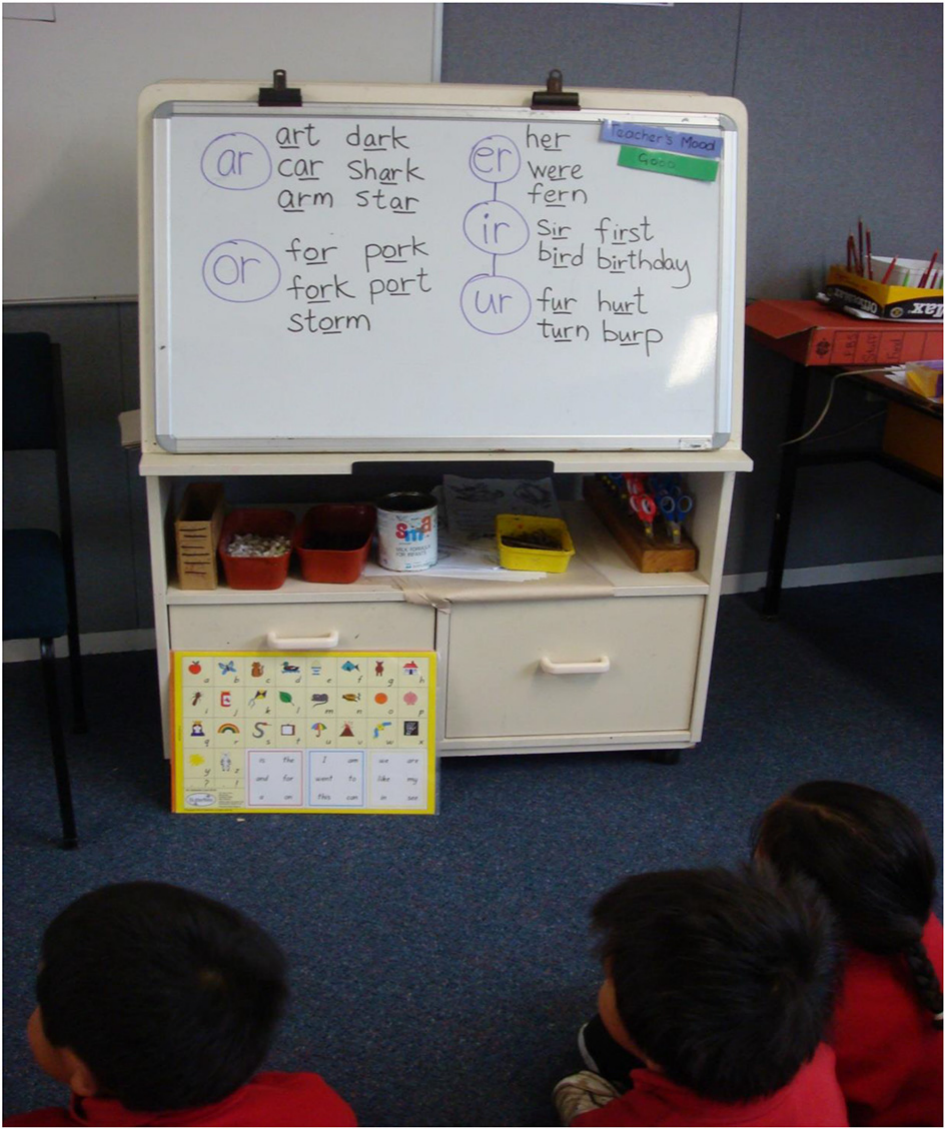
**A segment from a phonics lesson with word patterns written on the whiteboard to illustrate the sounds of r-affected vowels (ar, er, ir, or, ur)**.

***Phonics (P)***. Students learned and revised letter-sound rules for 25 min (Nicholson, 2005). The lessons followed the sequence of rules of Anglo-Saxon words in English (Calfee and Patrick, 1995)—Table 2 indicates the scope and sequence of phonics rules covered. Pupils were taught how to analyze printed words according to their sound patterns—for an example of phonics work during the lesson see Figure 2. There was no book reading in the lessons. Each lesson also included letter sound training based on the strategy of Turtle Talk (Gough and Lee, 2007). Turtle Talk involves stretching out the sounds in a word to make them more salient, e.g., “s-u-n.” The Turtle Talk activity involved the tutor saying the individual sounds in a word slowly, one after the other, with students attempting to guess the word. It was explained to pupils that turtle talk was a way of saying words slowly just as a turtle walks slowly. This activity is called Turtle Talk because the tutor talks slowly at the speed of a turtle, which was the hypothetical explanation given to pupils.

**Table 2:** **The scope and sequence of lesson plans for the combined group (1, first reading; 2, 2nd reading; 3, 3rd reading)**.

**Lesson**	**Lower**	**Middle**	**Higher**
1	Alphabet chart	Blends and digraphs	Silent e rule (lesson 1)
	Story: Car shopping (1st reading)	Story: Keep trying (1)	Story: The hole in the King's sock (1)
2	Consonant blends	Silent e (lesson 1)	Silent e rule (lesson 2)
	Story: Car shopping (2nd reading)	Story: Keep trying (2)	Story: The hole in the King's sock (2)
3	Vowels + silent e (lesson 1)	Silent e (lesson 2)	R-affected vowels
	Story: Car shopping (3rd reading)	Story: Keep trying (3)	Story: The hole in the King's sock (3)
4	Silent e (lesson 2)	R- affected vowels	L-affected vowels
	Story: What does Greedy Cat like? (1)	Story: Lunch for Greedy Cat (1)	Story: A good idea (1)
5	R-affected vowels	L-affected vowels	Vowel digraphs ai/ay and oi/oy
	Story: What does Greedy Cat like? (2)	Story: Lunch for Greedy Cat (2)	Story: A good idea (2)
6	L-affected vowels	Vowel digraphs ai/ay and oi/oy	ee
	Story: What does Greedy Cat like? (3)	Story: Lunch for Greedy Cat (3)	Story: A good idea (3)
7	Vowel digraphs ai/ay and oi/oy	ee	ie
	Story: Greedy Cat's door (1)	Story: Hissing Bush (1)	Story: Earthquake (1)
8	ee	ie	oa and ew
	Story: Greedy Cat's door (2)	Story: Hissing Bush (2)	Story: Earthquake (2)
9	ie	oa and ew	au and aw
	Story: Greedy Cat's door (3)	Story: Hissing Bush (3)	Story: Earthquake (3)
10	oa and ew	au and aw	ea
	Story: Keep Trying (1)	Story: Magnetic Max (1)	Story: Firefighter (1)
11	au and aw	ea	oo and ou
	Story: Keep Trying (2)	Story: Magnetic Max (2)	Story: Firefighter (2)
12	ea	oo and ou	Syllable breaking (CVC/CVC)
	Story: Keep Trying (3)	Story: Magnetic Max (3)	Story: Firefighter (3)

In addition to the oral language form of Turtle Talk the tutor also printed words on a whiteboard and pointed to the letters in the words while the pupils were turtle talking. The tutor was modeling how to decode words according to their letter-sounds. This was not part of the original Turtle Talk activity but was added to the lesson to get a message across to pupils that they can apply Turtle Talk to the decoding of words.

***Big Book (BB)***. The students in the Big Book only group read Big Books that were slightly above their instructional reading level—Table 2 shows the scope and sequence of Big Book lessons. Ten of the Big Books were published by the Ministry of Education and two by a commercial publisher. Big Books are almost 40 cm high and 30 cm wide in dimension, and illustrated with large print. The tutor used concepts and ideas from *Ready to Read: Teacher support materials* (Ministry of Education, 2001). Each story lasted for three reading sessions. During the lesson, the tutor read the Big Book several times to and with the students. The tutor read the text with the students as choral reading in the first and second reading. In the third reading, the tutor drew students' attention to one or two of the following areas: phonics (e.g., the gr for *greedy* in the *Greedy Cat* story), punctuation (e.g., speech marks, full stops, and capital letters), language features (e.g., opposites - *little* and *big, old* and *new*), or asked interactive questions after the reading about the overall meaning of the story including aspects of the text structure such as plot or character. Over the 12 weeks, the tutor read twelve different Big Books, that is, four different Big Books for each ability group.

***Big Books enhanced with explicit phonics (BB/EP)***. In the combined group, pupils covered the same Big Books as for the Big Books group and the same phonics and phonemic awareness lessons as for the phonics group but with less depth because it was a shorter time frame to do both sets of activities. Table 2 shows the scope and sequence of the 12 lessons for the combined group which covered the same phonics rules as the phonics group and the same Big Books as the Big Book group. The Appendix shows an example of a silent e lesson given to the combined Big Book/Phonics reading group where explicit phonics enhanced the Big Book reading (for examples of other BB/EP lessons from this study, see Nicholson and Dymock, 2014).

**Figure 3:**
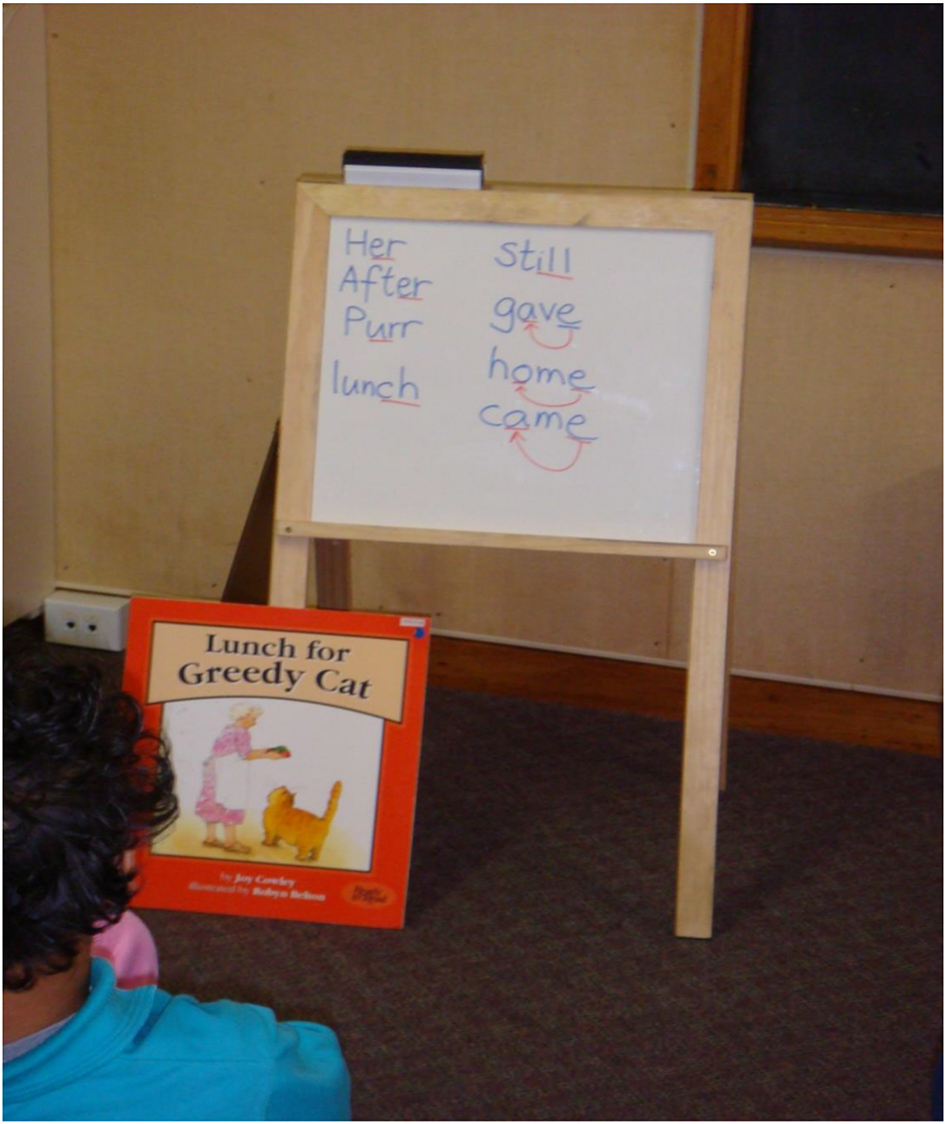
**A segment from a Big Book/Phonics combined lesson (BB/EP) with word patterns from the Big Book written on the whiteboard to illustrate the sounds of r-affected and l-affected vowels, and the silent e pattern**.

The tutor started the lesson with a decoding rule and worked on the Big Book that had examples of this rule. The scope and sequence was the same as for the phonics and Big Book lessons but the instruction for each was condensed so as to use both kinds of instruction. As with the phonics lessons, students in the three reading ability groups also engaged in Turtle Talk using words from the story. After the Turtle Talk activity, the tutor wrote on the whiteboard a short list of words that followed decoding rules. The task for students was to associate Turtle Talk phonemes spoken by the tutor with their written representations on the whiteboard. An example of phonics words taken from the Big Book is shown in Figure 3. The tutor wrote the words *her, after, purr, lunch, gave, home, came, and still* on the whiteboard. As in the phonics lessons, when doing the phonemic awareness activity the tutor asked students to listen carefully when she slowly said the sounds in the word, e.g., “keh-ay-m” (for *came*), to blend the sounds together in their minds, then to say the word aloud, and point to the correct answer on the whiteboard. Students also performed this activity in reverse (e.g., what word is “m-ay-keh”).

***Control group (M)***. Students in the control group received Math instruction and the same amount of instructional time as the other treatment groups. This condition controlled for placebo effects, that is, the effects of receiving special attention. Students learned about numbers and the quantities they stand for, specifically, counting, comparing numbers, addition, subtraction, and multiplication. All students practiced and computed math questions at different ability levels. For example, the lower ability reading group learned basic one-digit addition, the middle ability reading group learnt one- and two-digit addition, and the higher ability reading group learned at a more advanced level for addition.

#### Weekly quizzes

**Figure 4:**
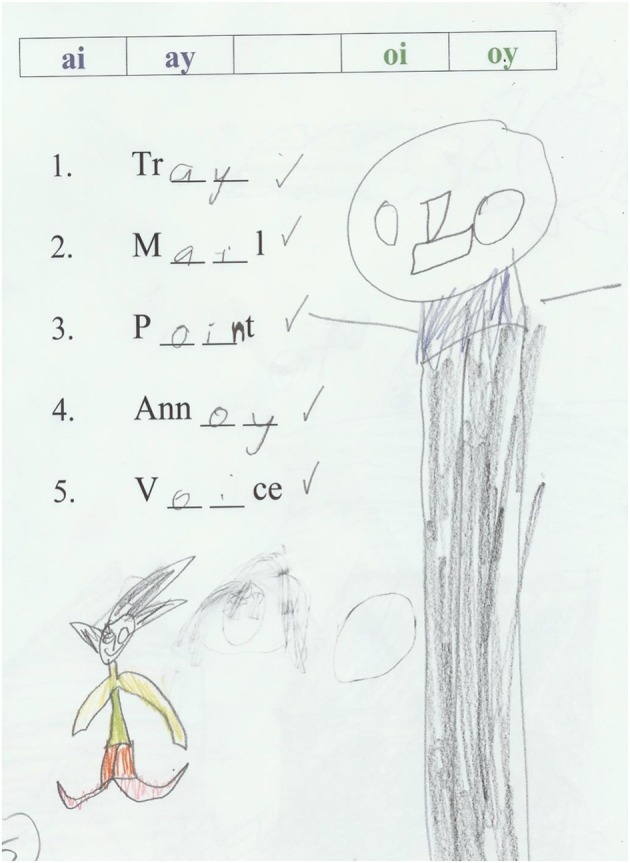
**Example of pupil answers for a quiz about the ai-ay and oi-oy phonics patterns**.

The purpose of having quizzes was to assess learning of phonics rules for the Phonics and Combined groups—see Table 3 for the scope and sequence of quizzes and Figure 4 for an example of a quiz. The quizzes were given to all four groups each week, at the end of each lesson, except for the first lesson. Each quiz had five questions. The paper-and-pencil quiz took 5 min to complete and tested different decoding patterns, for example, the silent *e* rule, consonant blends/digraphs, and vowel digraphs. The quizzes covered phonics rules taught in the BB/EP and Phonics group lessons with different quizzes for each reading ability group. The lower group were assessed on single letter sounds, consonant blends and digraphs, short vowel sounds as in *hop*, the split digraph rule (silent e) as in *hope*, r- and l-affected vowel sounds as in *car, wall*, and single-sound vowel digraphs such as ai, ay as in *rain* and *ray*. The middle group was assessed on similar rules but with an additional two-sound vowel digraph tested, ea as in *beach* and *bread*. The higher group was assessed on similar patterns to those of the lower and middle groups but with the addition of two-sound digraphs oo as in *book* and *roof* and ou as in *soup* and *mouse*.

**Table 3:** **Scope and sequence of the 10 quizzes for ability groups**.

**Quiz**	**Lower**	**Middle**	**Higher**
1	Single sounds^*^	Consonant blends and digraphs^*^	Short and long vowels/silent e [split digraphs]^a^^*^
2	Consonant blends and digraphs	Short and long vowels/silent e [split digraphs]^a^	Short and long vowels/silent e [split digraphs]^b^
3	Short and long vowels/silent e [split digraphs]^a^	Short and long vowels/silent e [split digraphs]^b^	r-affected vowels
4	Short and long vowels/silent e [split digraphs]^b^	r-affected vowels	l-affected vowels
5	r-affected vowels	l-affected vowels	ai-ay and oi-oy
6	l-affected vowels	ai-ay and oi-oy	ee and ie^**^
7	ai-ay and oi-oy	ee and ie^**^	oa and ew
8	ee and ie ^**^	oa and ew	au and aw
9	oa and ew	au and aw	ea
10	au and aw	ea	oo and ou

* *Quiz 1 started in Week 2 of the intervention*.

** *Lessons on ee and ie combined in one quiz*.

a,b *The silent e pattern is called a split digraph in England*.

#### Measures

***Word reading***. The Burt Word Reading Test (Gilmore et al., 1981) is a norm-referenced test standardized in New Zealand which assesses the ability to read words out of context. Students read words presented on a test card with 110 words printed in different sizes of type and graded in approximate order of difficulty from easy words like *to* and *big* to difficult words like *ingratiating* and *poignanc*y. In this test, students read as many words as they can and stop when they make 10 consecutive errors (or miscues). They then look over the remaining words to see if they recognize any other words. The test manual reports high test- retest reliability (*r* > 0.95) and high internal consistency (*r* > 0.96). The reason for using this test is that it is the only New Zealand norm-referenced word reading test standardized for use with 6-year-old pupils. The test-retest correlation in the current study was *r* = 0.86, *N* = 96.

***Passage reading***. The Neale Analysis of Reading-3rd Edition (Neale, 1999) is a norm-referenced test for pupils aged 6 to 12 years which has two parallel forms. The test assesses passage oral reading accuracy, ability to comprehend passages, and rate of reading. We did not assess rate of reading in this study mainly because children in the lower groups at pretest were reading hardly any words. Pupils completed the green form (Form 2) in the pretest and the yellow form (Form 1) in the posttest. Each form consisted of six passages graded in difficulty. The pupil reads the passages aloud and then answers comprehension questions asked by the examiner. Students cannot look back at the story when answering comprehension questions. The test has a high level of internal consistency with correlations ranging from 0.71–0.96. We chose this test because it is the only available norm-referenced measure for 6-year-olds that assesses reading accuracy and comprehension of passages with norms for a similar population to New Zealand (the test was standardized in Australia). The test-retest correlations for this measure were *r* = 0.88, *N* = 96 for accuracy and *r* = 0.67, *N* = 96 for comprehension.

***Basic decoding skills***. The Bryant Test of Basic Decoding Skills (Bryant, 1975; reprinted in Nicholson, 2005) is a list of 50 pseudowords read aloud by the student. The test starts with one-syllable consonant-vowel-consonant (CVC) combinations such as *buf*, then moves to silent-e patterns such as *fute*, consonant digraphs such as *thade*, vowel digraphs such as *groy*, and ends with multisyllabic pseudowords such as *vomazful*. Pupils had to pronounce the word correctly as a whole word, not just sounding out each letter. When students made 10 consecutive errors, testing stopped and students were encouraged to look at the rest of the list to check if they could read any other words. Juel (1988) reported reliabilities between 0.90 and 0.96 for this test. This test is not norm-referenced. We chose this test because it assessed basic decoding skills and because its scope and sequence of difficulty matched with phonics rules taught in the study (e.g., the pseudoword *fute* targeted knowledge of the silent e rule). The test-retest correlation in this study was *r* = 0.72, *N* = 96.

***Phonemic awareness***. The Gough-Kastler-Roper (GKR) Test of Phonemic Awareness (Roper, 1984; reprinted in Nicholson, 2005) has 42 items divided into six categories of seven items each assessing a different aspect of phonemic awareness: phonemic segmentation, blending, deletion of initial and final phonemes, and initial and final phoneme substitution. This is an oral assessment measure where students do not see the items. The assessor reads out the questions and the students respond to them verbally (e.g., what are the two sounds in “up”?). The assessor stops after 10 consecutive errors. Roper (1984) reported reliabilities greater than *r* = 0.7 for all subtests of this measure. This test is not norm-referenced. We chose this test because it has been successfully used in other New Zealand studies and it has a range of difficulty. The test-retest correlation in this study was *r* = 0.77, *N* = 96.

***Receptive vocabulary***. The British Picture Vocabulary Scale (BPVS II) (Dunn et al., 1997) is a norm-referenced receptive vocabulary assessment. For example, one of the test pages has four pictures: butterfly, baby, bed and shoe. The pupil points to the picture that represents the word spoken by the examiner (e.g., “bed”). There are 168 target words. The median reliability according to the examiner manual is 0.90. The reason for choosing this measure is that it is suitable for the age group and we wanted to know if the Big Book reading experience had a positive effect on vocabulary learning. The test-retest reliability in this study was *r* = 0.67, *N* = 96.

***Spelling***. The Schonell Spelling Test (Schonell, 1951) is a series of words graded in difficulty. The assessor says the word, says it in a sentence, and says the word again. The pupil then spells the word. The test starts with three-letter words (e.g., net, can, fun) and extends to multi-syllabic words (e.g., irresistible, hydraulic, anniversary). Stevenson et al. (1993) reported high reliability, *r* = 0.97 for the test. The test was suitable for use with young pupils in that the words slowly increase in difficulty. The test-retest correlation in this study was *r* = 0.84, *N* = 96.

***Math***. The WRAT 3 Wide Range Achievement Test (Wilkinson, 1993) is a norm-referenced test of math computation. The test divided into 2 parts. Part 1 was given orally with 15 questions involving counting, identifying numbers, and solving simple oral problems, such as “Read these numbers out loud” and “Which number is more, 9 or 6?” Part 2 was a pencil and paper test with 40 math problems, with questions suitable for this age group, such as 2+1 =, 5−3 =, 4× 2=. Students answered as many questions as they could in 15 min. Raw score is the number of questions answered correctly in parts 1 and 2 of the test. The test manual reported reliabilities from 0.87–0.96. We chose this test because it started with very simple calculations and it did not involve reading. The test-retest correlation in this study was *r* = 0.56, *N* = 96.

### Data analysis

The pre-post battery and the quizzes were both analyzed by standard factorial ANOVA techniques, augmented by orthogonal contrasts to assess specific questions for the Ability and Treatment factors. For Ability, orthogonal polynomials were used to evaluate the linear and quadratic trends across the three levels. For Treatment, Helmert contrasts (Keppel and Wickens, 2004), also orthogonal, served to answer the following questions from the research problem:

1. Does performance of the Math Control group (C) differ from the average of the other treatment groups?
2. Does performance of the Combined group (BB/EP) differ from the average of the two single-treatment groups?
3. Do the two single-treatment groups, Big Book (BB) and Phonics (P) differ from one another?

The analyses of all measures were based on *N* = 96 except for the spelling and basic decoding skills measures where for each measure one of the children did not complete the assessment as intended. For these measures the analyses were based on *N* = 95.

Effect sizes were measured using the partial omega square statistic (ω^2^) which suited the contrast analyses. Keppel and Wickens (2004) recommend this statistic as most suitable for orthogonal contrasts. Omega square statistics report the amount of variance accounted for by the contrast. A small effect captures about 1% of the variance, a medium effect about 6% of the variance and a large effect about 15% of the variance. Only the omega square statistics for each contrast are reported in Table 5 since these are the most important effects for this study.

## Results

**Figure 5:**
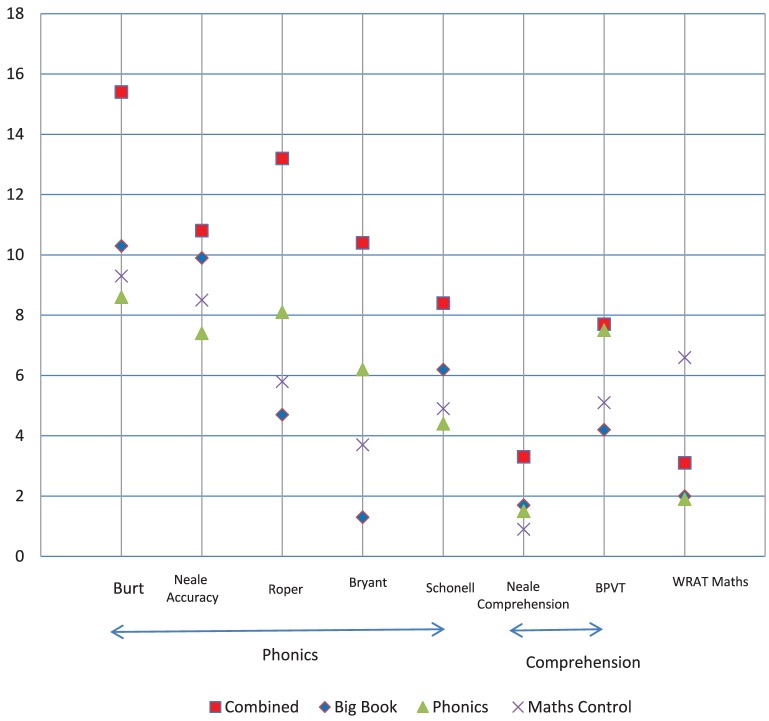
**The pre-post difference raw scores for the four treatment groups for each measure**.

**Figure 6:**
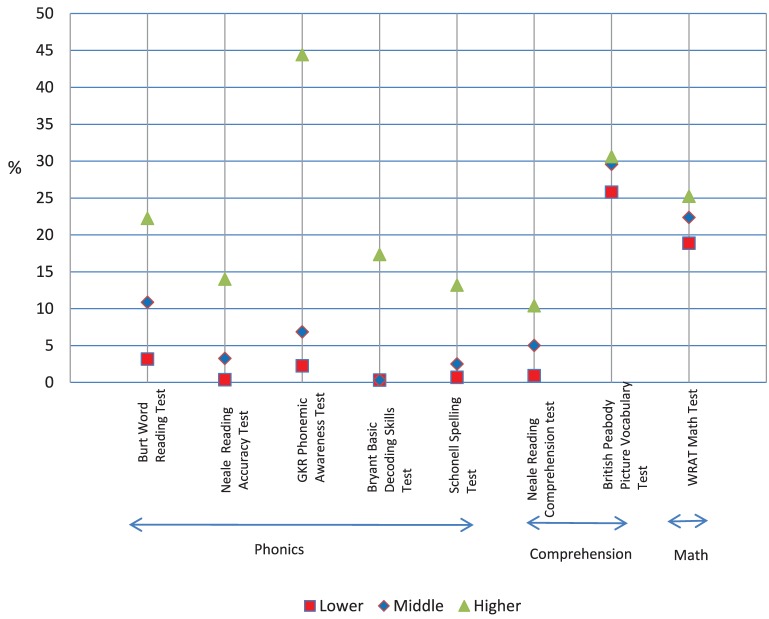
**The pretest scores for the three ability groups expressed as percentage of maximum score for each measure**.

**Figure 7:**
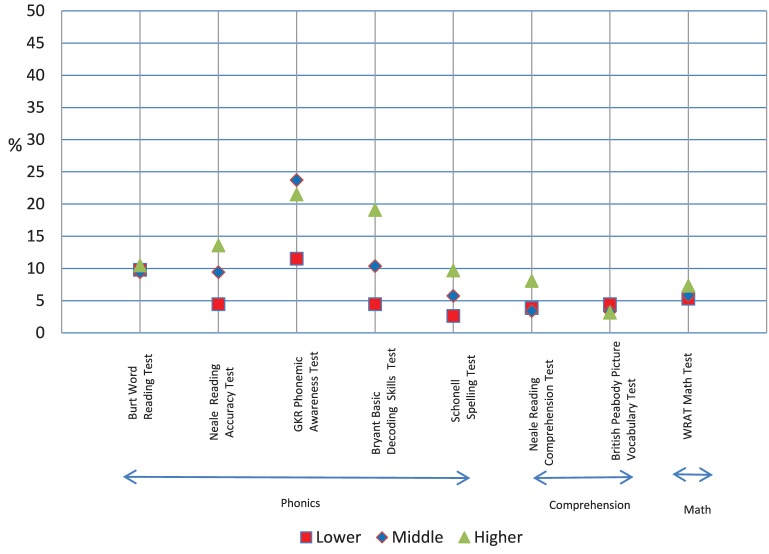
**The pre-post difference scores for the three ability groups expressed as percentage of maximum score for each measure**.

The pretest, posttest, and difference mean scores, and standard deviations for the eight dependent measures are shown in Table 4. The statistical analyses are shown in Table 5. The prepost difference raw scores for the four treatment groups are shown in Figure 5 to make comparisons clearer. The difference scores for ability are presented in Figures 6, 7 as percent scores in order to show trend differences with a common metric. The percent score was the difference score divided by the maximum score for each measure. We report the findings for the treatment groups first.

**Table 4:** **Descriptive statistics showing pretest, posttest, and prepost differences for higher, middle, and lower ability pupils in the control, combined, Big Book, and phonics training groups (with minimum and maximum scores indicated for each measure) (*N* = 96)**.

**Reading level**		**Math control**			**Combined**			**Big Book**			**Phonics**		
		**Pre-**	**Post-**	**Diff**	**Pre-**	**Post-**	**Diff**	**Pre-**	**Post-**	**Diff**	**Pre-**	**Post-**	**Diff**
**WORD READING (BURT)**													
**Min-Max (0–110)**													
Lower	*M*	3.4	14.6	**11.3**	2.5	19.4	**16.9**	4.1	11.6	**7.5**	4.0	11.5	**7.5**
	*SD*	4.2	6.3	5.7	2.2	5.7	5.4	3.4	5.0	2.4	3.5	8.1	6.4
Middle	*M*	12.0	20.6	**8.6**	11.6	26.9	**15.3**	13.8	25.0	**11.3**	10.5	16.9	**6.4**
	*SD*	4.7	5.8	3.8	5.4	5.8	3.5	6.6	4.9	5.6	5.2	5.8	6.3
Higher	*M*	23.3	31.4	**8.1**	22.8	36.8	**14.0**	24.6	36.6	**12.0**	27.3	39.3	**12.0**
	*SD*	8.3	9.7	5.7	7.2	6.8	2.6	6.3	7.7	5.3	3.7	8.6	8.2
Across level	*M*	12.9	22.2	**9.3**	12.3	27.7	**15.4**	14.2	24.4	**10.3**	13.9	22.5	**8.6**
	*SD*	10.1	10.1	5.1	9.9	9.3	4.0	10.1	11.9	4.9	10.8	14.3	7.2
**PASSAGE ACCURACY (NEALE)**													
**Min-Max (0–100)**													
Lower	*M*	0.6	6.1	**5.5**	0.1	5.3	**5.1**	0.1	3.3	**3.1**	0.6	4.6	**4.0**
	*SD*	1.8	4.8	3.9	0.4	5.4	5.2	0.4	3.2	3.2	1.2	5.6	4.9
Middle	*M*	4.1	12.4	**8.3**	3.1	14.6	**11.5**	3.9	16.1	**12.3**	1.9	7.5	**5.6**
	*SD*	4.1	7.7	4.9	2.9	6.0	4.4	4.2	3.9	3.3	1.9	4.5	3.7
Higher	*M*	12.1	23.9	**11.8**	12.9	28.6	**15.8**	14.5	28.9	**14.4**	16.5	29.0	**12.5**
	*SD*	9.2	13.4	4.6	10.1	10.1	2.8	8.2	8.9	5.5	9.5	9.5	9.4
Across level	*M*	5.6	14.1	**8.5**	5.4	16.2	**10.8**	6.2	16.1	**9.9**	6.3	13.7	**7.4**
	*SD*	7.5	11.7	5.0	8.0	12.1	6.0	8.0	12.1	6.4	9.1	12.9	7.2
**PASSAGE COMPREHENSION (NEALE)**													
**Min-Max (0–36)**													
Lower	*M*	0.6	1.3	**0.6**	0.3	2.3	**2.0**	0.4	1.8	**1.4**	0.1	1.6	**1.5**
	*SD*	1.1	1.4	1.2	0.5	1.2	1.1	1.1	1.8	2.0	0.4	1.2	1.3
Middle	*M*	2.0	2.5	**0.5**	1.5	4.4	**2.9**	1.4	3.0	**1.6**	2.4	2.3	**−0.1**
	*SD*	1.2	2.0	1.4	1.4	2.1	2.4	0.9	1.5	1.4	1.4	1.2	1.0
Higher	*M*	3.5	5.0	**1.5**	3.5	8.4	**4.9**	4.1	6.1	**2.0**	3.9	7.1	**3.3**
	*SD*	2.5	3.9	3.0	1.8	2.1	1.6	2.0	2.2	2.5	2.9	3.7	3.2
Across level	*M*	2.0	2.9	**0.9**	1.8	5.0	**3.3**	2.0	3.6	**1.7**	2.1	3.7	**1.5**
	*SD*	2.0	3.0	2.0	1.9	3.1	2.1	2.1	2.6	1.9	2.4	3.4	2.4
**PHONEMIC AWARENESS (ROPER)**													
**Min-Max (0–42)**													
Lower	*M*	1.4	3.4	**2.0**	0.1	10.4	**10.3**	2.3	6.5	**4.3**	0.0	2.9	**2.9**
	*SD*	2.9	4.3	2.2	0.4	5.4	5.4	4.5	7.9	7.4	0.0	2.5	2.5
Middle	*M*	1.5	11.0	**9.5**	5.4	22.1	**16.8**	1.5	6.1	**4.6**	3.1	12.1	**9.0**
	*SD*	2.0	11.2	10.4	8.0	11.2	8.7	2.8	3.8	4.1	3.6	9.4	6.1
Higher	*M*	15.8	21.8	**6.0**	19.6	32.1	**12.5**	22.0	27.1	**5.1**	17.3	29.8	**12.5**
	*SD*	14.1	10.4	9.6	9.0	5.1	6.2	9.2	9.3	10.5	15.4	8.6	9.1
Across level	*M*	6.2	12.0	**5.8**	8.4	21.5	**13.2**	8.6	13.3	**4.7**	6.8	14.9	**8.1**
	*SD*	10.6	11.7	8.5	10.7	11.7	7.1	11.3	12.2	7.5	11.6	13.4	7.4
**BASIC DECODING SKILLS (BRYANT)**													
**Min-Max (0–50)**													
Lower	*M*	0.5	1.9	**1.4**	0.0	5.8	**5.8**	0.1	0.1	**0.0**	0.0	1.8	**1.8**
	*SD*	1.4	3.2	3.2	0.0	7.5	7.5	0.4	0.4	0.5	0.0	3.3	3.3
Middle	*M*	0.0	3.1	**3.1**	0.1	12.9	**12.8**	0.1	1.0	**0.9**	0.4	4.4	**4.0**
	*SD*	0.0	3.2	3.2	0.4	6.2	6.0	0.4	2.1	2.1	0.7	5.3	5.3
Higher	*M*	9.4	16.0	**6.6**	6.9	19.5	**12.6**	10.1	13.3	**3.1**	9.5	22.3	**12.8**
	*SD*	12.9	15.4	5.7	6.9	4.9	3.6	7.4	7.6	10.7	9.8	11.1	7.7
Across level	*M*	3.3	7.0	**3.7**	2.3	12.7	**10.4**	3.5	4.8	**1.3**	3.3	9.5	**6.2**
	*SD*	8.4	11.0	4.6	5.0	8.3	6.6	6.3	7.5	6.2	7.0	11.7	7.3
**SPELLING (SCHONELL)**													
**Min-Max (0–100)**													
Lower	*M*	1.9	3.1	**1.3**	0.1	5.3	**5.1**	0.6	1.6	**1.0**	0.1	3.3	**3.1**
	*SD*	5.3	5.2	2.9	0.4	5.5	5.5	1.8	2.6	1.4	0.4	4.4	4.3
Middle	*M*	2.1	8.9	**6.8**	3.0	12.1	**9.1**	0.9	7.0	**6.1**	4.0	4.4	**0.4**
	*SD*	2.5	7.2	5.7	4.7	4.1	5.9	2.1	4.7	4.0	3.5	4.6	3.6
Higher	*M*	14.3	20.9	**6.6**	12.5	23.5	**11.0**	13.1	24.6	**11.5**	12.9	22.5	**9.6**
	*SD*	12.6	13.7	5.8	8.4	7.5	3.8	7.1	8.6	7.1	6.5	5.6	5.8
Across level	*M*	6.1	11.0	**4.9**	5.2	13.6	**8.4**	4.9	11.1	**6.2**	5.7	10.0	**4.4**
	*SD*	9.7	11.7	5.4	7.6	9.5	5.5	7.3	11.5	6.3	6.8	10.2	6.0
**BRITISH PEABODY VOCABULARY TEST (BPVT)**													
**Min- Max (0–168)**													
Lower	*M*	44.5	52.3	**7.8**	41.0	49.6	**8.6**	45.4	50.3	**4.9**	42.5	51.3	**8.8**
	*SD*	11.4	14.6	9.6	3.7	7.2	6.8	10.9	6.8	12.2	6.3	8.0	9.0
Middle	*M*	55.1	59.1	**4.0**	50.1	56.0	**5.9**	45.1	52.9	**7.8**	48.4	53.3	**4.9**
	*SD*	5.9	9.6	6.9	12.2	12.2	9.8	10.2	9.0	7.7	9.6	8.7	7.0
Higher	*M*	49.3	52.8	**3.5**	52.1	60.8	**8.6**	50.9	50.9	**0.0**	53.5	62.4	**8.9**
	*SD*	14.2	10.3	8.6	10.6	5.3	12.1	5.9	7.8	9.4	6.6	11.0	8.2
Across level	*M*	49.6	54.7	**5.1**	47.8	55.5	**7.7**	47.1	51.3	**4.2**	48.1	55.6	**7.5**
	*SD*	11.5	11.6	8.3	10.4	9.6	9.5	9.3	7.7	10.0	8.6	10.2	8.0
**MATHEMATICS (WRAT 3)**													
**Min-Max (0–55)**													
Lower	*M*	10.3	15.6	**5.4**	11.5	13.8	**2.3**	10.3	12.8	**2.5**	9.5	11.0	**1.5**
	*SD*	3.3	4.1	3.2	2.6	2.4	2.1	1.5	2.2	2.3	2.1	1.7	2.7
Middle	*M*	12.8	20.6	**7.9**	12.8	15.4	**2.6**	11.1	12.3	**1.1**	12.6	13.8	**1.1**
	*SD*	1.8	2.1	3.6	1.5	2.2	2.6	2.2	3.5	3.6	2.0	2.8	3.2
Higher	*M*	14.3	20.8	**6.5**	12.5	16.9	**4.4**	14.4	16.6	**2.3**	14.4	17.4	**3.0**
	*SD*	2.7	2.3	1.8	2.4	1.6	1.8	2.6	4.5	2.5	2.2	1.6	2.2
Across level	*M*	12.4	19.0	**6.6**	12.3	15.3	**3.1**	11.9	13.9	**2.0**	12.2	14.0	**1.9**
	*SD*	3.0	3.8	3.0	2.2	2.4	2.3	2.7	3.9	2.8	2.9	3.3	2.8

**Table 5:** **Results of Three-Way ANOVAs for pretest and prepost difference data for each measure with polynomial contrasts for ability and helmert contrasts for group, using a random effects general linear model and partial omega square effect sizes**.

		**Pretest**		**Prepost difference**	
**Variables**	***df***	***F***	***ω^2^***	***F***	***ω^2^***
**BURT WORD READING**					
Ability	2	117.50^**^		0.41	
Linear	1	232.26^**^	**0.78**	0.34	0.00
Quadratic	1	2.86	0.03	0.49	0.00
Group	3	0.62		8.37^**^	
Math/other	1	0.20	0.00	2.92	0.04
Combined/BB,P	1	1.61	0.00	20.98^**^	**0.29**
BB vs. phonics	1	0.03	0.00	1.19	0.00
Ability × group	6	0.54		1.81	
In group team	12	0.61		1.42	
*MS* error	72	(30.31)		(26.84)	
**NEALE ACCURACY**					
Ability	2	47.34^**^		35.36^**^	
Linear	1	85.19^**^	**0.57**	70.56^**^	**0.52**
Quadratic	1	9.49^**^	**0.12**	0.17	0.00
Group	3	0.14		2.89^*^	
Math/other	1	0.06	0.00	0.71	0.00
Combined/BB,P	1	0.35	0.00	3.88^#^	**0.06**
BB vs. phonics	1	0.01	0.00	4.08^*^	**0.06**
Ability × group	6	0.49		1.47	
In group team	12	0.63		2.98^**^	
*MS* error	72	(34.87)		(19.01)	
**NEALE COMPREHENSION**					
Ability	2	33.67^**^		7.15^**^	
Linear	1	67.08^**^	**0.51**	9.61^**^	**0.12**
Quadratic	1	0.42	0.00	4.67^*^	**0.05**
Group	3	0.22		6.25^**^	
Math/other	1	0.06	0.00	7.56^**^	**0.12**
Combined/BB,P	1	0.49	0.00	11.16^**^	**0.17**
BB vs. phonics	1	0.12	0.00	0.05	0.00
Ability × group	6	0.38		1.33	
In group team	12	0.48		1.11	
*MS* error	72	(2.77)		(3.89)	
**PHONEMIC AWARENESS**					
Ability	2	45.89^**^		3.97^*^	
Linear	1	76.21^**^	**0.54**	4.67^*^	**0.05**
Quadratic	1	15.52^**^	**0.18**	3.28	0.03
Group	3	0.50		5.67^**^	
Math/other	1	0.79	0.00	2.37	0.03
Combined/BB,P	1	0.12	0.00	12.25^**^	**0.19**
BB vs. phonics	1	0.58	0.00	0.05	0.03
Ability × group	6	0.48		0.85	
In group team	12	0.37		0.37	
*MS* error	72	(65.87)		(59.99)	
**SPELLING**					
Ability	2	40.17^**^		19.83^*^	
Linear	1	69.06^**^	**0.51**	39.44^*^	**0.38**
Quadratic	1	11.22^**^	**0.14**	0.18	0.00
Group	3	0.19		3.60^**^	
Math/other	1	0.34	0.00	2.04	0.02
Combined/BB,P	1	0.00	0.00	7.18^**^	**0.11**
BB vs. phonics	1	0.22	0.00	1.56	0.01
Ability × group	6	0.24		2.09	
In group team	12	0.47		2.18^*^	
*MS* error	71	(36.21)		(20.21)	
**BASIC TEST OF DECODING SKILLS (NON-WORDS)**					
Ability	2	22.22^**^		20.89^**^	
Linear	1	33.52^**^	**0.34**	41.34^**^	**0.39**
Quadratic	1	11.36^**^	**0.14**	0.50	0.00
Group	3	0.15		14.38^**^	
Math/other	1	0.26	0.00	5.90^*^	**0.09**
Combined/BB,P	1	0.36	0.00	29.05^**^	**0.37**
BB vs. phonics	1	0.02	0.00	8.29^**^	**0.13**
Ability × group	6	0.11		1.81	
In group team	12	0.17		2.57^**^	
*MS* error	71	(33.91)		(20.37)	
**BRITISH PEABODY VOCABULARY TEST**					
Ability	2	6.31^**^		0.52	
Linear	1	11.42^**^	**0.14**	0.90	0.00
Quadratic	1	1.23	0.00	0.14	0.00
Group	3	0.30		0.82	
Math/other	1	0.76	0.00	0.38	0.00
Combined/BB,P	1	0.00	0.00	0.62	0.00
BB vs. phonics	1	0.13	0.00	1.46	0.01
Ability × group	6	0.92		0.67	
In group team	12	0.83		0.52	
*MS* error	72	(91.96)		(89.13)	
**MATHEMATICS**					
Ability	2	19.76^**^		1.52	
Linear	1	39.44^**^	**0.38**	2.79	0.03
Quadratic	1	0.15	0.00	0.23	0.00
Group	3	0.21		16.18^**^	
Math/Other	1	0.34	0.00	45.43^**^	**0.48**
Combined/BB,P	1	0.14	0.00	2.99	0.04
BB vs. phonics	1	0.15	0.00	0.01	0.00
Ability × group	6	1.63		1.11	
In group team	12	1.36		1.11	
*MS* error	72	(4.98)		(7.24)	

* p < 0.05;

** p < 0.01;

# *p = 0.053*.

*Math, control group; Combined, Big Book enhanced with phonics; Other, Combined, Big Book and Phonics; BB, Big Book; P, Phonics; In Group Team, small groups*.

### Group results for pretest and for pre-post difference scores

At pretest there were no significant group effects not as main effect or as a contrast. This showed that the treatment groups were equivalent at pretest. The results for the pre-post difference scores showed a different pattern altogether. In presenting the pre-post difference results we focus on the questions relating to the contrasts since they were most important in terms of the analysis.

Question 1. Does performance of the Math Control group differ from the average of the other treatment groups (C vs. BB/EP, BB, P)? As can be seen in Table 5 and Figure 5, the contrast between the control group and the other groups (Math/Other) for the language and literacy measures were sometimes not significant mainly due to the control group scoring more highly than the phonics and Big Book groups so that the average of the three groups was similar to the control group. The exceptions were two significant Math/Other effects for reading comprehension and basic decoding skills where the math group scored significantly below the average of the other treatment groups.

The control group (Math/Other) contrast was highly significant for the math measure and with a substantial effect size showing that the control group performed much better than the average of the three reading groups. This was because the control group received alternative math instruction and the other groups did not.

Question 2. Does the performance of the Combined group (BB/EP) differ from the average of the Big Book (BB) and Phonics (P) groups? As shown in Table 5 and Figure 5, the BB/EP group had significantly higher scores than the average mean score of the BB and P groups for word reading, reading comprehension, basic decoding skills, phonemic awareness and spelling. Two of the effect sizes were substantial (word reading and basic decoding skills). For reading accuracy, the BB/EP group was not significantly different to the average mean of the BB and P groups though it was nearly so [*p* = 0.053: BB/EP mean(diff) = 10.8, BB mean(diff) = 9.6, *P* mean(diff) = 7.4]. There was no significant effect for the contrast of the BB/EP group and the other two groups in relation to the vocabulary and math measures.

Question 3. Did the two single-treatment groups differ from one another? As shown in Table 5 and Figure 5, the final contrast between the BB and P groups showed a mixed picture for reading accuracy and decoding. For reading accuracy the BB group performed better than P and had similar scores to the BB/EP group. Thus, for reading accuracy we can infer that the BB/EP and BB groups made similar progress. For decoding the P group performed better than the BB group and had similar scores to the BB/EP group. Thus, we can infer that for basic decoding skills the BB/EP and P groups made similar progress. On all other measures (word reading, reading comprehension, phonemic awareness, spelling, vocabulary, and math) there was no difference between the BB and P groups.

**Figure 8:**
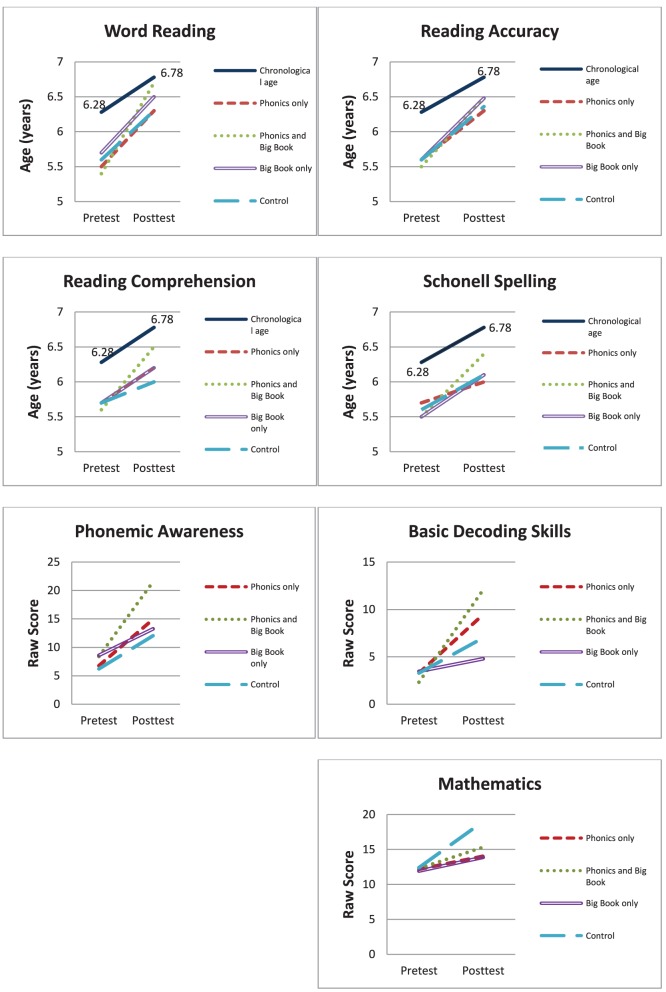
**Mean scores for each of the significant results for each of the training groups pretest to posttest**. Mean Burt and Neale results reported as reading ages; spelling reported as spelling age. Other results reported as raw scores.

To summarize the pre-post results for the treatment groups, the Combined BB/EP instruction was more effective than Big Book reading for all literacy measures except reading accuracy where there was no difference between the Combined and Big Book groups. Combined instruction was more effective than phonics for all literacy measures except basic decoding skills where it was equally effective. The control group who received math instruction made significantly more progress in math than the other three groups who did not receive math teaching. In Figure 8 the results for word reading, reading accuracy, and reading comprehension are expressed as reading ages and spelling as a spelling age to give a more meaningful interpretation of the results. These graphs show that for reading comprehension, word reading, and spelling, the BB/EP instruction brought the reading and spelling ages of these children closer to their chronological age. For reading accuracy, BB/EP and BB instruction both moved children closer to their chronological age.

### Ability results at pretest and for pre-post difference scores

#### Pretest

A trend analysis of pretest scores for word reading, reading comprehension, receptive vocabulary and math showed that the linear coefficient made a significant contribution in explaining the trend (effect sizes were from 0.14 to 0.78) but the quadratic coefficient did not (effect sizes 0.00 to 0.03). As can be seen in Figure 6, mean percent score (percent of maximum possible score) decreased similarly in line with reading ability (word reading: high = 22%, middle = 11%, low = 3%; reading comprehension: high = 10%, middle = 5%, low = 1%; vocabulary: high = 31%, middle = 30%, low = 26%; math: high = 25%, middle = 22%, low = 19%).

A trend analysis of pretest scores for reading accuracy, phonemic awareness, spelling, and basic decoding skills, showed that the linear coefficient (effect sizes were from 0.34 to 0.57) and quadratic coefficient (effect sizes were from 0.12 to 0.18) both made a significant contribution in describing the trend of the data, though the linear trend accounted for most of the variance. Although students' scores did decrease in a linear way from the higher group to the middle group, this pattern did not continue for the lower group. As can be seen in Figure 6, the middle and lower groups had similar percent scores that were well below those of the higher group (reading accuracy: high = 14%, middle = 3%, low = 1%; phonemic awareness: high = 44%, middle = 7%, low = 2%; spelling: high = 13%, middle = 2%, low = 1%; decoding skills: high = 17%, middle = 1%, low = 1%).

#### Posttest

A trend analysis of prepost gain scores for reading accuracy, phonemic awareness, spelling, and basic decoding skills showed that the linear coefficient made a significant contribution to explaining the trend (effect sizes were from 0.05 to 0.52) and that the quadratic coefficient did not (effect sizes were from 0.00 to 0.03). As can be seen in Figure 7, mean percent gains decreased similarly in line with reading ability (reading accuracy: high = 14%, middle = 9%, low = 4%; phonemic awareness: high = 21%, middle = 24%, low = 11%; spelling: high = 10%, middle = 6%, low = 3%; decoding: high = 19%, middle = 10%, low = 4%).

A trend analysis of gains for reading comprehension showed that the linear coefficient (effect size was 0.12) and the quadratic coefficient (effect size was 0.05) both made a significant contribution to explaining the trend. As can be seen in Figure 7, there was a linear decrease in prepost comprehension gain from the higher to middle group but this pattern did not continue for the lower group whose percent gain was similar to that of the middle group (reading comprehension: high = 8%, middle = 3%, low = 4%).

A trend analysis of prepost difference scores for word reading, receptive vocabulary, and math showed no significant linear or quadratic trends. As can be seen in Figure 7, the three ability groups made similar percent gains for these measures (word reading: high = 10%, middle = 9%, low = 10%; receptive vocabulary: high = 3%, middle = 3%, low = 4%; math: high = 4%, middle = 6%, low = 5%).

### Interactions

Pretest scores showed no significant ability × group interactions, indicating that the treatment groups were equivalent in ability at pretest. Prepost difference scores (gains) showed no significant ability × group interactions, indicating that the three ability groups made similar gains across the four treatment groups.

### In group team effects

There were no significant in-group team effects at pretest, indicating that the subgroup teams were equivalent. For pre-post measures there were significant in-group team effects for reading accuracy, spelling and basic decoding skills, indicating some differences among the subgroups. These were random effects, however, and not the focus of this design.

### Phonics quizzes

All groups completed the 10 weekly phonics quizzes. Each quiz had five questions and was marked out of 5. The marks for the 10 different quizzes were averaged to be out of 5 (see Table 6 for means and standard deviations). Each ability group did different quizzes. The analysis was the same ANOVA design as for the test battery except that it was not possible to include ability as a fixed effects factor because each ability group received different quizzes to match their ability level. The ANOVA results are shown in Table 7.

**Table 6:** **Average quiz scores: Means and Standard Deviations**.

**Reading level**		**Control (Math)**	**Combined (BB/EP)**	**Big Book**	**Phonics**
Lower	*M*	1.39	2.51	1.40	2.16
	*SD*	0.72	0.45	0.45	1.87
Middle	*M*	1.79	2.64	2.21	1.84
	*SD*	0.70	0.78	0.66	0.81
Higher	*M*	2.26	2.79	2.35	2.48
	*SD*	1.20	1.37	0.93	0.82
Across levels	*M*	1.81	2.65	1.99	2.16
	*SD*	1.20	1.37	0.93	0.82

**Table 7:** **Average quiz scores: separate ANOVAs for Lower, middle, and higher reading ability groups**.

**Variables**	***df***	***F***	**ω^2^**
**READING ABILITY**			
**Lower**			
Group	3	9.08^**^	
Math/other	1	8.70^**^	0.32
Combined/BB,P	1	10.18^**^	0.36
BB vs. phonics	1	8.35^**^	0.31
In group team	4	0.57	
*MS* error	24	(0.28)	
**Middle**			
Group	3	2.80^#^	
Math/other	1	2.66	0.09
Combined/BB,P	1	4.49^*^	0.18
BB vs. phonics	1	1.28	0.02
In group team	4	2.68	
*MS* error	24	(0.44)	
**Higher**			
Group	3	0.33	
Math/other	1	0.35	0.00
Combined/BB,P	1	0.58	0.00
BB vs. phonics	1	0.05	0.00
In group team	4	0.58	
*MS* error	24	(1.30)	

* p < 0.05;

** p < 0.01;

# *p = 0.06*.

*Math, control group; Combined, Big Book enhanced with phonics; Other, Combined, Big Book and Phonics; BB, Big Book; P, Phonics; In Group Team, small groups*.

The results for the lower reading ability group showed that the contrast between the control group and the average of the means of the other groups (Math/Other) was significant. The control group mean was considerably below the other groups. The contrast between the combined BB/EP group and the average of the other two reading groups was significant. Inspection of the mean scores showed that the BB/EP group was higher than the other groups. The contrast between Big Books and Phonics means scores was significant, showing that the Phonics group scores were higher than those of the Big Book group.

The results for the middle group showed that the contrast between the control group and the average of the means of the other groups (Math/Other) was not significant. The control group had the lowest score of the four groups but the Phonics group also had a similarly low score and this probably made the difference non-significant. The contrast between BB/EP and the average mean of the Big Book and Phonics groups was significant. The contrast between Big Books and Phonics means was not significant. From this we can infer that the combined BB/EP group had a higher mean score than did the other two reading groups.

The results for the higher ability group were not significant for any of the three contrasts. This indicated that the treatments did not have differential effects for the higher ability group.

In summary, inspection of the mean scores in Table 6 confirm the ANOVA results showing that for the lower ability group, the combined BB/EP and Phonics groups had significantly better quiz scores than the Big Book and control groups. For the middle ability reading group the combined group had better quiz scores than the other three groups. For the higher ability group, quiz scores were not significantly different among the four treatment groups.

## Discussion

The model that drove this study was that combining Big Book reading with explicit phonics would have benefits across the board for a range of literacy skills, more so than Big Book reading or explicit phonics on their own. This is what the study found. The findings highlight the importance of combining necessary skills with authentic reading experience to increase literacy achievement for disadvantaged children.

The current study cuts new ground in our understanding of the impact of Big Book reading and phonics on children's literacy development. While many studies have compared Big Book (or shared book) reading with phonics none to our knowledge have compared Big Books enhanced with explicit phonics (BB/EP) with Big Book reading or phonics on their own. Many experienced researchers, such as Pressley (2006), have concluded, based on their reading of the research for each kind of instruction, that balanced instruction using both practices must be more effective than either on their own. This study is the first to show that this conclusion is correct.

### The literacy gap

A relevant question for this study was whether the treatments were closing the reading ability gap, that is, whether they were increasing the learning rate for the lower/middle ability groups relative to the higher reading ability group. This did not happen. There was no interaction between treatments and reading ability for any of the measures. The lower reading ability groups did not outpace the higher reading ability group in relative gains for any of the treatment groups. Future research could look at refinements to the present study that might help to close the literacy gap.

### Specific results

#### Word reading

The Combined group did better than the other groups including the control group. In relation to the Big Books and Phonics groups this may have been because of the explicit phonics being applied to particular words from the Big Book text as part of the combined lessons (see the Appendix sample lesson). The combined instruction showed children how to use explicit phonics to help them decode words from their books. Children could see the practical application of phonics to reading in that the lessons would cover phonics aspects of some words from the Big Books before the teacher and the children began to read the Big Books. This focus on words from the books was not addressed in the Big Books group except in an incidental way and was not addressed at all in the Phonics group.

#### Reading accuracy

The Combined group did as well as the Big Books group in passage reading accuracy and better than the Phonics and control groups. The results for the control group are explainable in that they did not receive reading instruction. A possible explanation for the phonics group results is that the combined group and the Big Books group both engaged in Big Book reading whereas the explicit phonics group did not engage in reading of text. Thus, the phonics group did not get the opportunity to apply their skills to book reading. Research on phonics indicates that teaching skills in isolation without opportunities to apply these skills while reading will not help them improve in book reading (Compton et al., 2014).

#### Reading comprehension

The Combined group did better than the Big Books and Phonics groups in reading comprehension and this may have been because the explicit phonics in the combined instruction improved the word reading skills of children (as can be seen in their improved Burt word reading results) which in turn made the comprehension process easier by enabling the combined group children to focus more of their mental energy on comprehending what they read. There is support for this idea from other research (Tan and Nicholson, 1997) showing that improved word reading skills in a trained group produced better reading comprehension compared with a control group even though there was no difference in passage reading accuracy between the two groups. In other words the combined group did better than the other groups in comprehension because their superior word reading skills enabled them to process words more easily thus releasing more cognitive resources for comprehension.

#### Phonemic awareness

The Combined group made better progress in phonemic awareness than the other groups. This was understandable for the Big Books group in that they did not receive any instruction in phonemic awareness. A possible explanation why the combined group did better than the Phonics group who also received phonemic awareness instruction might be that using the Turtle Talk strategy to learn phonemic awareness in the combined group lessons may have been more effective because the phonemic training was on words from the Big Book stories they had read and this may have been more impacting in terms of learning how to read words when reading compared with the phonics phonemic exercises which were on unrelated words that were not part of book reading.

#### Basic decoding skills

The Combined and Phonics groups made similar progress in basic decoding skills and made better progress than did the Big Books group. This was understandable in that Big Book reading allows for incidental phonics learning but does not teach basic decoding skills in detail except to make use of initial consonant blends.

#### Spelling

The Combined group made better progress than did the other groups. This was understandable for the control group and Big Books group who received no explicit instruction in spelling though the Big Books group may have picked up spelling skills implicitly through reading of Big Books. The phonics group did learn skills useful for spelling but these words may not have been stored as well in memory as compared with the combined group because the words covered in the phonics lessons were not part of a Big Book whereas with the combined group the spelling activities involved words from a Big Book and these words may have been more memorable in terms of storing their component letters in memory.

#### Vocabulary

There were no differences among the four groups in receptive vocabulary. It was understandable that there would have been few gains in vocabulary for the phonics and control groups because they did not receive instruction in vocabulary. However there was the possibility that the Combined and Big Books groups might have improved vocabulary since they both focused on meaning and there is a strong body of research to indicate that reading books aloud to pupils improves vocabulary (McBride-Chang, 2012). The reason for the lack of an effect on vocabulary for the Combined and Big Books groups might have been that the Big Book lessons did not have enough complex vocabulary or there might not have been enough discussion of unfamiliar words. To address this issue, future research could look at the effects of adding activities that build more vocabulary and general knowledge instruction into the combined and Big Book lessons (Nicholson and Dymock, 2010; Compton et al., 2014).

#### Math

The results for the control group in math showed that small group instruction in mathematics had significant benefits for them in their learning of math skills as compared with the other groups in the study who did not receive this instruction. It was understandable that the other groups would not make similar gains because they received no math instruction. The math result was the strongest in the whole study. In hindsight it would have been interesting to combine math instruction with Big Book reading to see if this would also improve math skills. There are a number of children's books that have a math aspect to them and these could have been used to teach computation. Future research could look at this possibility.

## Conclusion

The findings reveal that we do not have to teach disadvantaged children in an either-or fashion, using either Big Book reading or phonics but we can combine the instruction, integrating them in a meaningful way, and produce better readers and spellers. If teachers included explicit phonics in their Big Book lessons even on a once-weekly basis, the present results indicate that this would have greater long-term benefits across more literacy measures than would Big Book reading or explicit phonics instruction on their own.

The big picture was that the combined instruction was as effective as Big Books for reading accuracy and was superior to Big Books for word reading, reading comprehension, spelling, basic decoding skills, and phonemic awareness. Likewise, the combined instruction was as effective as explicit phonics for basic decoding skills and was superior to phonics for all other measures of literacy.

To conclude, the present study found that Big Books enhanced with phonics, as compared with Big Book reading and phonics on their own, seemed to have no disadvantages and considerable advantages across a range of literacy measures. This type of balanced instruction could be a model for New Zealand and other countries wanting to find more effective ways to teach literacy to disadvantaged children, who are the ones we are very concerned about.

### Conflict of interest statement

The authors declare that the research was conducted in the absence of any commercial or financial relationships that could be construed as a potential conflict of interest.

## Appendix

Lesson plan example – Lesson 1 for higher reading ability pupils, Combined group (BB/EP), 1st reading of Big Book The Hole in the King's Sock, phonics rule was silent e.

Introduction

1. Focus: Silent e rule
2. Story: *The Hole in the King's Sock* (Level-Orange)

Teacher (T): Hello, we are going to learn a rule which is called the silent e rule, and then read the story about the King who found a hole in his sock.

T: Do you know what a vowel is? In English, we have 5 letters with vowel sounds and each letter stands for two sounds, one long and one short. First of all, the 5 vowels are written as: a, e, i, o, u and sometimes y is also included as well. The sounds of the vowels usually change when there is an e at the end of the word, and we call this the silent e rule. (Then recap that the short sounds are the actual sounds of the letters and the long sounds are the names of the letters).

Lesson: (using whiteboard)

T: The silent e rule for *a_e* means that when you see the word spelled *ate* it says “ate”- the special e makes the vowel says its name. I am going to underline the vowels.

T: Remember, the letter e is silent in “ate*”*, and this e is going to make the other vowel “ay” say its name. Let's say this word together.

T: Well done! Let's have a look at some other examples on the whiteboard.

**Table:**

Short vowel	Long vowel
mat	mate
hat	hate
pet	Pete
pin	pine
cop	cope
cut	cute

T: Let's have a look at these words from the story - you are going to see them in the story later.

**Table:**

c**a**m**e**	g**a**v**e**	m**a**d**e**
wove	stitched	dough
knit	knitting	wriggled
gold	learn	thread

Students look at the word as a whole first, sounding them out if they do not know the word. They repeat and read the words 2 times.

**Table:**

came: c-a-me (silent e)	gave: g-a-ve (silent e)
made: m-a-de (silent e)	wove: w-o-ve (silent e) past tense of weave
stitched: st-i-tch-ed (tch =“ch” sound)	d-ough: irregular word
knit: kn-i-t (silent k)	knitting: kn-i-tt-i-ng
wriggled: wr-i-gg-l-ed (silent w)	g-o-l-d
l-ear-n	th-r-ea-d

Activity: Turtle Talk (researcher selects 5–6 words from chart above)

The students listen to the phonemes of the words provided by the researcher e.g., “m-ay-deh” and they have to point out the correct word on the whiteboard. Pupils get a chance to Turtle Talk and say the word and the teacher has to guess what it is.

The teacher explains the silent e rule again when reading words from the story that had the silent e pattern - *came, gave, made, wove*. The word *dough* from the story is an irregular word. The -tch in *stitched* has the ch sound because ch is spelled tch after a short vowel sound. Explain that *knit* and *knitting* both have a silent k; *wriggled* has a silent w.

T: Great, I am going to read you the story of *The Hole in the King's Sock*, and I am going to ask you some questions about what happened in the story afterwards. Before we start, what are socks? Yes, they are covers we put on our feet. Where do you buy your socks from?

Pupils say: the warehouse, the supermarket, two-dollar shop.

T: Well, we will see what happens to the King's sock. Now, please listen carefully to the story (during the reading, encourage students to predict what might happen next).

Comprehension questions (orally)

1. What was the King's problem?
2. Did he find a solution? What was that?
3. Did it work? If not, why did it not work?
4. Was the problem solved at the end?
